# Multimodal AI for prediction of distant metastasis in carcinoma patients

**DOI:** 10.3389/fbinf.2023.1131021

**Published:** 2023-05-09

**Authors:** Isaac Olatunji, Feng Cui

**Affiliations:** Thomas H. Gosnell School of Life Science, Rochester Institute of Technology, Rochester, NY, United States

**Keywords:** metastasis, cancer, multimodal, gene expression, histopathology, deep learning, machine learning

## Abstract

Metastasis of cancer is directly related to death in almost all cases, however a lot is yet to be understood about this process. Despite advancements in the available radiological investigation techniques, not all cases of Distant Metastasis (DM) are diagnosed at initial clinical presentation. Also, there are currently no standard biomarkers of metastasis. Early, accurate diagnosis of DM is however crucial for clinical decision making, and planning of appropriate management strategies. Previous works have achieved little success in attempts to predict DM from either clinical, genomic, radiology, or histopathology data. In this work we attempt a multimodal approach to predict the presence of DM in cancer patients by combining gene expression data, clinical data and histopathology images. We tested a novel combination of Random Forest (RF) algorithm with an optimization technique for gene selection, and investigated if gene expression pattern in the primary tissues of three cancer types (Bladder Carcinoma, Pancreatic Adenocarcinoma, and Head and Neck Squamous Carcinoma) with DM are similar or different. Gene expression biomarkers of DM identified by our proposed method outperformed Differentially Expressed Genes (DEGs) identified by the DESeq2 software package in the task of predicting presence or absence of DM. Genes involved in DM tend to be more cancer type specific rather than general across all cancers. Our results also indicate that multimodal data is more predictive of metastasis than either of the three unimodal data tested, and genomic data provides the highest contribution by a wide margin. The results re-emphasize the importance for availability of sufficient image data when a weakly supervised training technique is used. Code is made available at: https://github.com/rit-cui-lab/Multimodal-AI-for-Prediction-of-Distant-Metastasis-in-Carcinoma-Patients.

## Introduction

Proper management planning for carcinoma patients requires accurate diagnosis, and prognosis prediction. A major prognostic factor, metastasis, however is not always diagnosed at initial patient presentation. Metastasis refers to the dissemination of cancer cells away from the initial site of origin to form colonies at distant organs. This single hallmark of malignancies is responsible for the highest proportion (approximately 90%) of cancer related mortalities (Fares et al., 2020). In HNSCC (Head and Neck Squamous Cell Carcinoma), about 10% of patients present with Distant Metastasis (DM) at diagnosis, while 20%–25% are detected during the disease course (Pisani et al., 2020). Pancreatic cancer is usually diagnosed at a late stage, and DM is quite common at presentation, however, multidetector computed tomography (MDCT) which is currently the optimal preoperative investigation has poor sensitivity to liver and peritoneal metastasis, the most common metastatic sites (Liu et al., 2018). Cases of metastatic non-muscle-invasive bladder cancer have also been reported (Xu et al., 2022). Early diagnosis of metastatic cancer is critical if patients will benefit from systemic therapies, alongside other appropriate management plans. The mechanisms behind metastasis, however, are very complex, and a lot is still not understood. Currently, there are no standard biomarkers of metastasis (Wang et al., 2018), and identification of biomarkers that are associated with metastasis will be useful in guiding clinical decisions, and as basis for development of new therapies.

Past studies have investigated the presence or absence of generalizable links between genes involved in the metastasis of different cancer types, either on the basis of mutation, or expression level. However, metastasis has proven to be a complex molecular and biochemical process. While similar mutation rate, and expression pattern have been reported in selected genes across groups of metastatic cancer, there are multiple claims that there are in fact no specific cancer metastatic genes. In a study carried out by Liu et al. (2017), mutation rates of *TP53* was significantly different between primary and metastatic samples in seven cancer types, while *PTEN* mutation level was different in five cancer types. Copy number variations also differ significantly in all 15 cancer types examined. Nguyen et al. (2022) also implicated *TP53, PTEN, CDKN2A, and MYC* as significantly mutated genes in the metastasis of various subsets of cancer types.

In specific cancer types, designated differentially expressed signature genes are the basis of some of the past attempts to stratify cancer patients based on the risk of DM (van de Vijver et al., 2002). A recent study (Kaur et al., 2022) carried out in triple negative breast cancer samples using DESeq2 software identified a total of 1738 differentially expressed genes between metastatic and non-metastatic primary samples, 3 of which are part of the 70 prognostic signature genes in (van de Vijver et al., 2002). An analogous study in renal cell carcinoma noted some of the identified differentially expressed genes as predictive of metastasis-free survival, and overall survival (Ho et al., 2017). In melanoma, measurement of Breslow’s thickness of the primary tumor was correlated with the level of expression of specific genes, and the transition to metastatic tumor (Riker et al., 2008). While all of these reports suggest that genomic data may actually be predictive of metastasis, the inconsistent patterns of gene mutation and expression seen in different cancer samples makes it a challenge to precisely identify their importance in specific cases.

The wealth of morphological information contained in the tumor microenvironment is routinely exploited by pathologists for making definitive diagnosis of cancer and predicting patient prognosis. However, aside from being time consuming, this tedious process has also been associated with inter- and intra-observer variability that sometimes lead to unresolved diagnosis or worse, errors in diagnosis. With the use of Convolutional Neural Network (CNN), histopathology images have proven to be good predictors of malignancy status, important molecular biomarkers of various clinical and research relevance, as well as other cellular and extracellular processes (Mungenast et al., 2021). While many deep learning models have achieved relatively high metrics in detecting tumors within histopathology images of metastatic lymph node samples (Chuang et al., 2021; Wen et al., 2021; Huang et al., 2022), the difficult problem of predicting DM from primary samples remains a challenge, and most of the past attempts on this and similar tasks have struggled with relatively average model performance (Zhao, 2020; Brinker et al., 2021; Kiehl et al., 2021; Schiele et al., 2021).

Recent trends of use of multimodal data for prediction of diagnosis and outcomes in cancer patients have reported mostly improved metrics compared to use of singular modes of data (Mobadersany et al., 2018; Chen et al., 2022). In the case of metastasis prediction, past works have used singular or multimodal data combining clinical (Ali, 2020), genomic (Yuan et al., 2019), radiological (Liu et al., 2020), and histopathology (Zhao, 2020) data, however, to the best of our knowledge, at the time of this writing, this is one of the first works that combines transcriptomic data, clinical data, and histopathology images from primary tumor samples to predict DM.

In this work, we attempt to predict DM using gene expression data, clinical data, and histopathology images from primary tumors of three carcinoma types - Pancreatic Adenocarcinoma (PAAD), Bladder Carcinoma (BLCA), and Head and Neck Squamous cell Carcinoma (HNSC). The contributions of this research include:

- We identify genomic markers of DM in three different carcinoma types using a novel combination of the random forest algorithm with an optimized feature selection approach described in (Mori et al., 2021). Genes selected via this method performed better in prediction of DM than differentially expressed genes (DEGs) derived from DESeq2 analysis in our study, as well as in comparison to methods from other similar studies. These biomarkers could be further investigated for development of new diagnostics and therapies against DM.
- Using various machine learning techniques, we investigate and substantiate claims that genes involved in DM tend to be more cancer type specific rather than general across all cancer types, and that there are no specific cancer metastasis genes.
- We built separate models to predict DM from gene expression data, clinical data, or histopathology images, as well as multimodal combinations of the three data types. Models metrics show that multimodal data provides an edge for prediction of DM over genomic, clinical or histopathology data. However, the genomic data has the highest contribution by a wide margin.
- Unlike with genomic data in which features tend to be more cancer type specific, models built from histopathology image dataset of all three cancer types in total had better metrics than those built from a single cancer type image dataset, emphasizing the importance of sufficient data to build a robust model when a weakly supervised technique is used.

## Methods

### Dataset

Barcode of patients with DM in the TCGA-HNSC, TCGA-BLCA, and TCGA-PAAD projects were retrieved from Genomic Data Commons (GDC) Data Portal, and gene expression data, clinical data, and histopathology images of these patients were downloaded. The few number of available cases of DM is a common challenge in most studies on metastasis. BLCA, and PAAD were selected based on the number of cases of DM in these cancer types that are available on The Cancer Genome Atlas (TCGA), and the number of cases with available corresponding genomic, image, and clinical data. A few cancer types were dropped during this selection process due to the absence of some variables in their records. For example, Skin Cell Carcinoma (SKCM) was eliminated due to missing “number_of_lymph_nodes_positive_by_HE” variable which is present in BLCA, PAAD, and HNSC datasets. HNSC was added based on the interest of one of the authors. See Supplementary Material S1 for file ID and barcode of samples. BLCA (N = 80) records had 59 male, and 21 female patients. Average age in PAAD (N = 58; Male = 32; Female = 26), and HNSC(N = 24; Male = 21; Female = 3) records is 63, and 61 respectively. The race of patients in the BLCA cohort are (White = 66; Black or African American = 6; Asian = 5), and (White = 47; Black or African American = 3; Asian = 5) for PAAD, while HNSC had (White = 19; Black or African American = 4; Asian = 1). The T, and N staging, and Number of lymph nodes positive by hematoxylin and eosin (HE) staining in each cancer type is shown in Table 1.

**TABLE 1:** Information about metastatic datasets.

Information	Bladder Carcinoma	Pancreatic adenocarcinoma	Head and Neck Cancer
Number of samples	80	58	24
Patient age range	Min: 47	Min: 41	Min: 49
	Max: 90	Max: 81	Max: 79
Gender	Male: 59	Male: 32	Male: 21
	Female: 21	Female: 26	Female: 3
Race	White: 66	White: 47	White: 19
	Black or African American: 6	Black or African American: 3	Black or African American: 4
	Asian: 5	Asian: 5	Asian: 1
T staging	T1: 0	T1: 2	T1: 2
	T2: 26	T2: 7	T2: 4
	T3: 41	T3: 48	T3: 6
	T4: 13	T4: 1	T4: 12
N staging	0: 42	0: 17	0: 4
	1: 16	1: 41	1: 2
	2: 19	2: 0	2: 18
	3: 3	3: 0	3: 0
Number of lymph nodes positive by HE	0: 21	0: 16	0: 3
	1: 12	1: 5	1: 2
	2–6: 13	2–6: 28	2–6: 11
	>6: 8	>6: 8	>6: 3
	NA/Invalid: 26	NA/Invalid: 1	NA/Invalid: 5

### Machine learning for identification of transcriptomic biomarkers and prediction of distant metastasis

We designed a study that utilized machine learning techniques to simultaneously identify biomarkers of DM in each of BLCA, PAAD, and HNSC, and investigate if genes involved in DM are similar across the three cancer types. Each cancer type dataset was pre-processed separately. Equal numbers of complementary gene expression data of samples without DM (BLCA = 80; PAAD = 58; HNSC = 24) were downloaded for each of the cancer types, and we ensured that there were no overlapping samples between the groups with DM, and those without DM. After extracting Transcript Per Million (TPM)) normalized values of protein-coding genes from the individual records, all samples in a group were merged to create a table, and NaN values were replaced with zero. Other initial preprocessing steps include log to base ten transformation of data to adjust for the wide and non-linear values, removal of genes with a value of zero in greater than 80% of the samples, and selection of only top 10000 genes with highest variance across all samples.

**FIGURE 1:**
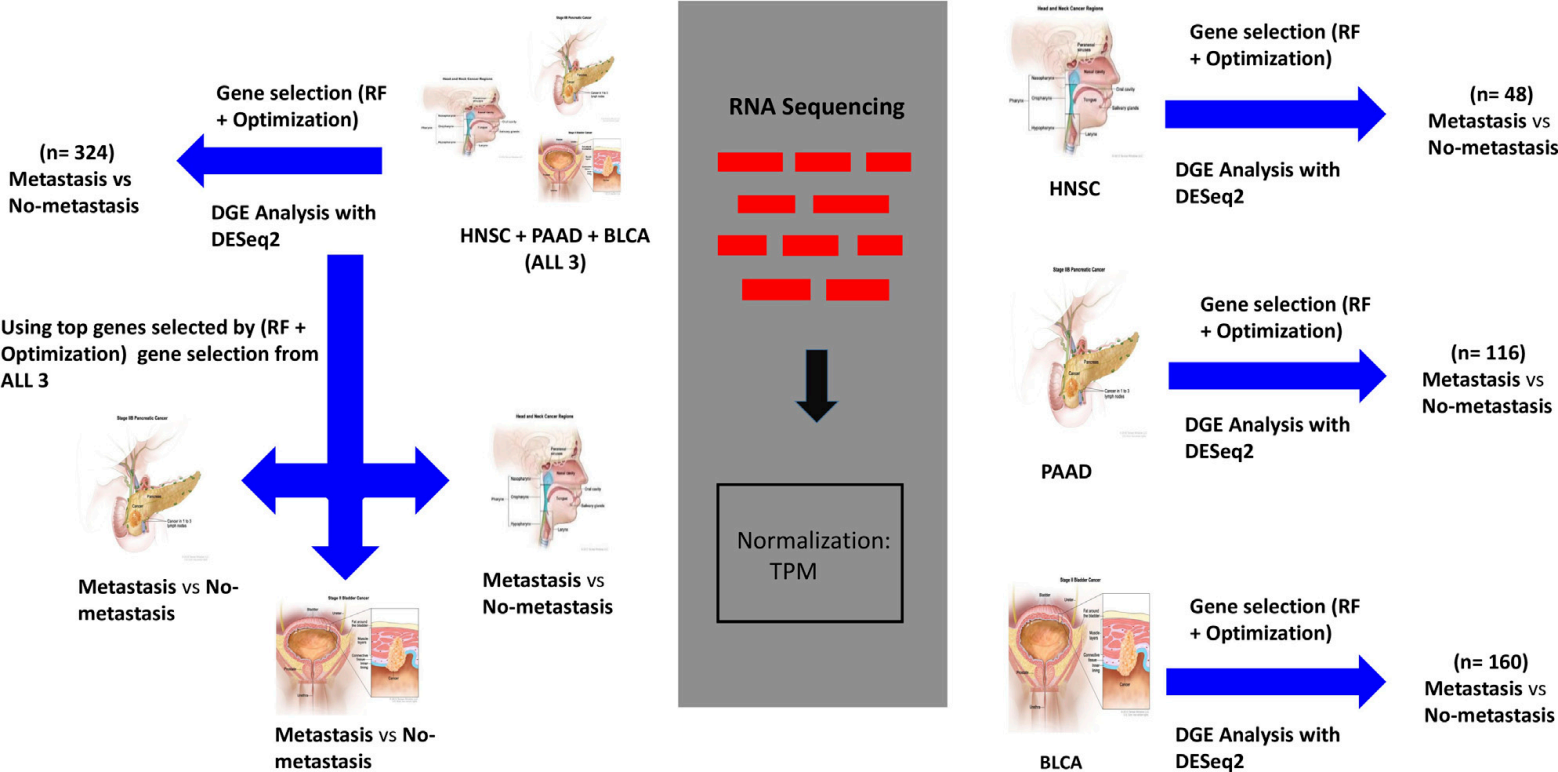
Workflow for analysis of genomic data. On the right, gene expression datasets of HNSC, PAAD, and BLCA are separately passed through the (RF + Optimization) gene selection algorithm, and a parallel DGE Analysis. Separate ML models are trained on highest ranked selected genes, and DEGs to predict DM samples. On the top left, the same processes carried out on ALL3 (combined dataset of HNSC, PAAD, and BLCA) dataset. On the lower left, genes selected from ALL3 dataset are used as features to train new models on each of the cancer types (i.e., other groups- HNSC, PAAD, BLCA) dataset. Cancer images source: National Cancer Institute.

To identify important genes that are involved in DM, we utilized the Random Forest (RF) algorithm with an optimization technique described in (Mori Y. et al., 2021). RF is a supervised ML algorithm that creates multiple decision trees from bootstrapped samples data and randomly selected features to output a result, that is, based on a voting system. It works well with high dimensional data, and is a commonly utilized technique in gene expression data analysis. The optimization technique we employed consists of running the RF algorithm over (N = 1000) iterations to classify samples as DM positive or DM negative, and selecting the top (K = 100) most important genes at each of the N instances. This is followed by ranking the important genes overall based on how many times each gene appears (i.e., frequency) in K over the N iterations. The algorithm was used to classify samples in each of the groups based on the presence or absence of DM (Figure 1). We selected the top 50 overall highest ranked genes for BLCA, PAAD, and HNSC. Also, the three cancer types datasets were combined to derive the “ALL3” group, and the above steps were repeated to select the top 50 highest ranked genes in this group as well (Figure 1).

To assess the strength of the selected biomarkers for prediction of DM, and to investigate if genes involved in DM are similar across the three cancer types, first, we used only the selected genes in each of the groups as features to train multiple ML models for the task of DM prediction. Support Vector Machine (SVM), K-Nearest Neighbor (KNN) and RF models were trained on datasets from each of the four (BLCA, PAAD, HNSC ALL3) groups (Figure 1), and the metrics were evaluated. Next, we looked for overlaps between different combinations of the selected genes in the four groups. Furthermore, we trained new ML (SVM, RF, and KNN) models on BLCA, PAAD, and HNSC datasets, but with genes selected from the combined dataset of the three cancer types (i.e., ALL3 group) (Figure 1). Lastly, we created a list of union of selected genes from each of the three cancer types (i.e., BLCA + PAAD + HNSC), and these were used as variables to predict presence or absence of DM in each of the three cancer dataset.

### Differential Gene Expression with DESeq2

To further assess the strength of genes selected by the proposed method for predicting DM, under the same study design, we carried out an analysis to identify Differentially Expressed Genes (DEGs) between samples with DM and those without DM in each of the four groups. Instead of the TPM normalized values, raw counts of unstranded RNASeq were extracted from the TCGA RNASeq records. A sample information table was also generated. Differential Gene Expression (DGE) analysis was performed in R using the DESeq2 Bioconductor package. DESeq2 detects DEGs by normalizing raw count values of genes in the experimental groups, fitting negative binomial generalized linear models for each gene, and detecting significance by Wald test (Love et al., 2014). Threshold was set at p-adjusted value of 0.05, after initial set threshold of log fold-change of 1 and p-adjusted value of 0.05 yielded only five, one and seven DEGs in BLCA, PAAD and HNSC respectively. Thereafter, top DEGs were used as features to classify DM samples in each of the study groups (Figure 1). DEGs from this analysis and metrics of models trained on them are compared to those of genes selected via the (RF + Optimization) method.

### Convolutional neural network for histopathology images analysis and prediction of distant metastasis

We downloaded diagnostic pathology Whole Slide Histopathology Images (WSIs) of patients with DM in the TCGA projects of BLCA, HNSC, and PAAD from GDC Data Portal. The number of samples in each group reduced slightly after collating a list of only patients with available histopathology images, and RNASeq data, and clinical data. Number of samples with DM in BLCA, PAAD, and HNSC groups are 50, 44, and 18 respectively. Again, we downloaded random complementary WSIs (BLCA = 55; PAAD = 51; HNSA = 22) of patients without DM for each of the cancer types, and ensured no overlap between samples (Supplementary Material S1). Total number of samples in each group was split in an 80:20 ratio for models training and testing.

### WSIs preprocessing

One WSI was preprocessed per patient. Due to the typical large size of WSIs which hovers around 100000 pixels in both horizontal and vertical axis, we isolated representative regions within each WSI for analysis. First, a maximum of 500 random non-overlapping patches of size 512 * 512 pixels were extracted from each image at 20 × magnification. These were reduced to a maximum of 200 patches after checking for a tissue area of at least 80% in each patch. To ensure selection of tumor representative regions we imitated the concept of high cellularity described in (Riasatian, 2021) which assumes that high grade tumors contain more cellular areas than normal tissues. Hence, we used a pretrained U-Net nuclei segmentation model which was trained on breast cancer histopathology images to rank the patches based on cellular content. The top 60 highest ranked patches were selected for each patient. Further random manual inspection led to the exclusion of a few more patches before Macenko normalization. At the end of initial preprocessing steps, BLCA, PAAD, HNSC, and ALL3 groups had a total of 4936, 4348, 1856, and 11154 training patches respectively, of which 25% were for validation.

### CNN training and multimodal fusion of genomic and imaging data

**FIGURE 2:**
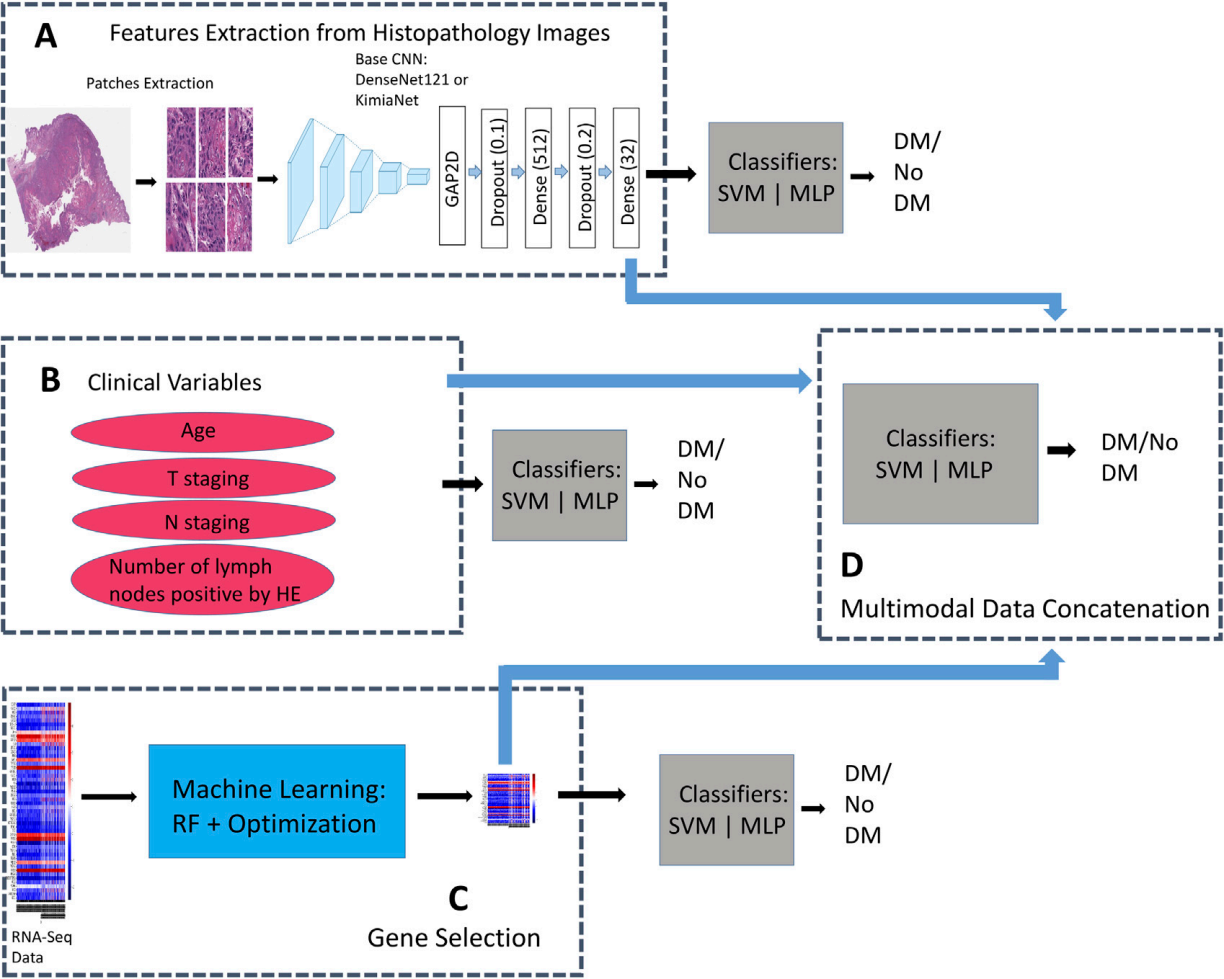
Binary classifiers trained on the same patients’ records but with different data types for comparisons between unimodal, and multimodal data models. **(A)** Additional Dense layers trained on DenseNet121, and KimiaNet CNN for features extraction from histopathology images. Classifiers are trained on image features to predict presence or absence of DM. **(B)** Classifiers are trained on only clinical variables to predict presence or absence of DM. **(C)** Classifiers are trained on genes selected by the (RF + Optimization) algorithm to predict presence or absence of DM **(D)** Image features are fused with selected genes, and clinical features to train multimodal models for prediction of DM.

A DenseNet121 model, pretrained on Imagenet data, and KimiaNet (Riasatian, 2021) were chosen for this study. This allows us to evaluate the effect of keeping weights of lower layers of a model fine-tuned on domain histopathology images (i.e., KimiaNet) on performance. DenseNet is a CNN architecture that attempts to solve the problem of varnishing gradient associated with deep neural networks by cumulatively concatenating features output of a layer within the architecture to input of subsequent upper layers, (Huang et al., 2016; 2017). In total, DenseNet121 contains 1 7 × 7 convolution, 58 3 × 3 convolution, 61 1 × 1 convolution, 4 average pooling, and 1 fully connected layers. KimiaNet is a DenseNet121 architecture Imagenet-pretrained CNN model fine-tuned on about 250,000 histopathology images. We replaced the classification layers in the models with 1 GlobalAveragePooling2D, 3 Dropout (0.1, 0.2, and 0.05), and 3 Dense (512, 32, and 1) layers, to train a new classifier head on top of the base convolutional layers, using a weakly supervised technique. The last hidden layer of the new classifier heads has 32 nodes which is subsequently used as features extractor. The modified DenseNet121 was separately trained on data from each of the four study groups (HNSC, BLCA, PAAD, ALL3) for a binary classification task of DM prediction **(** Figure 2 **)**. Loss function was binary cross-entropy, and optimizer, Adam with a learning rate of 0.0001. Training epoch was set at 40.

For prediction on the test sets, patient level features were derived by passing all patches for each patient through the trained feature extractor models, and averaging all patch features from a slide. We trained traditional machine learning algorithm- SVM and a single layer MLP on the patient level features to classify samples as DM positive or DM negative (Figure 2). Considering this approach we have taken, HNSC group was dropped here based on small sample size.

Multimodal concatenation of patient level features derived from the CNN models to log transformed values of TPM normalized RNASeq data of selected DM associated genes from above was carried out. For BLCA, and PAAD groups, the 50 genes selected using the (RF + Optimization) technique were combined with 32 features derived from histopathology images of each patient to train classifiers, and predict presence or absence of DM (Figure 2). 150 genes, consisting of 50 each selected from BLCA, PAAD, and HNSC were combined with 32 image features to predict DM in the ALL3 group (Figure 2).

Likely due to the effect of a larger training dataset given the weakly supervised training technique that was employed, it was observed from early results that image only models trained on data from the ALL3 group generally performed better in their predictions on the test sets than other groups. Similarly, multimodal models of genomic and histopathology image were generally better than unimodal model of either data type in the ALL3 dataset, while metrics of multimodal models are generally lower than either genomic or image only models in other individual cancer dataset (i.e., BLCA, or PAAD). Therefore further experiments were carried out on the ALL3 dataset. We trained fully connected layers on KimiaNet, a DenseNet121 architecture model finetuned on about 250,000 histopathology images to extract features from the images in the ALL3 dataset. Metrics of multimodal models built from these features, and genomic data were compared to those from DenseNet121.

Four variables—Age, T stage, N stage, and Number of lymph nodes positive by HE—were selected from the clinical data, and concatenated with genomic, and histopathology features to predict presence or absence of DM in the ALL3 dataset (Figure 2). As part of the clinical data preprocessing, invalid/NaN values in the age variable were replaced by the rounded mean age of the cancer type while invalid/NaN T staging, N staging and number of positive lymph node values were replaced with 0. Min-max normalization was applied to age, and number of positive lymph node variables.

## Results

### ML identified gene expression biomarkers of distant metastasis

**FIGURE 3:**
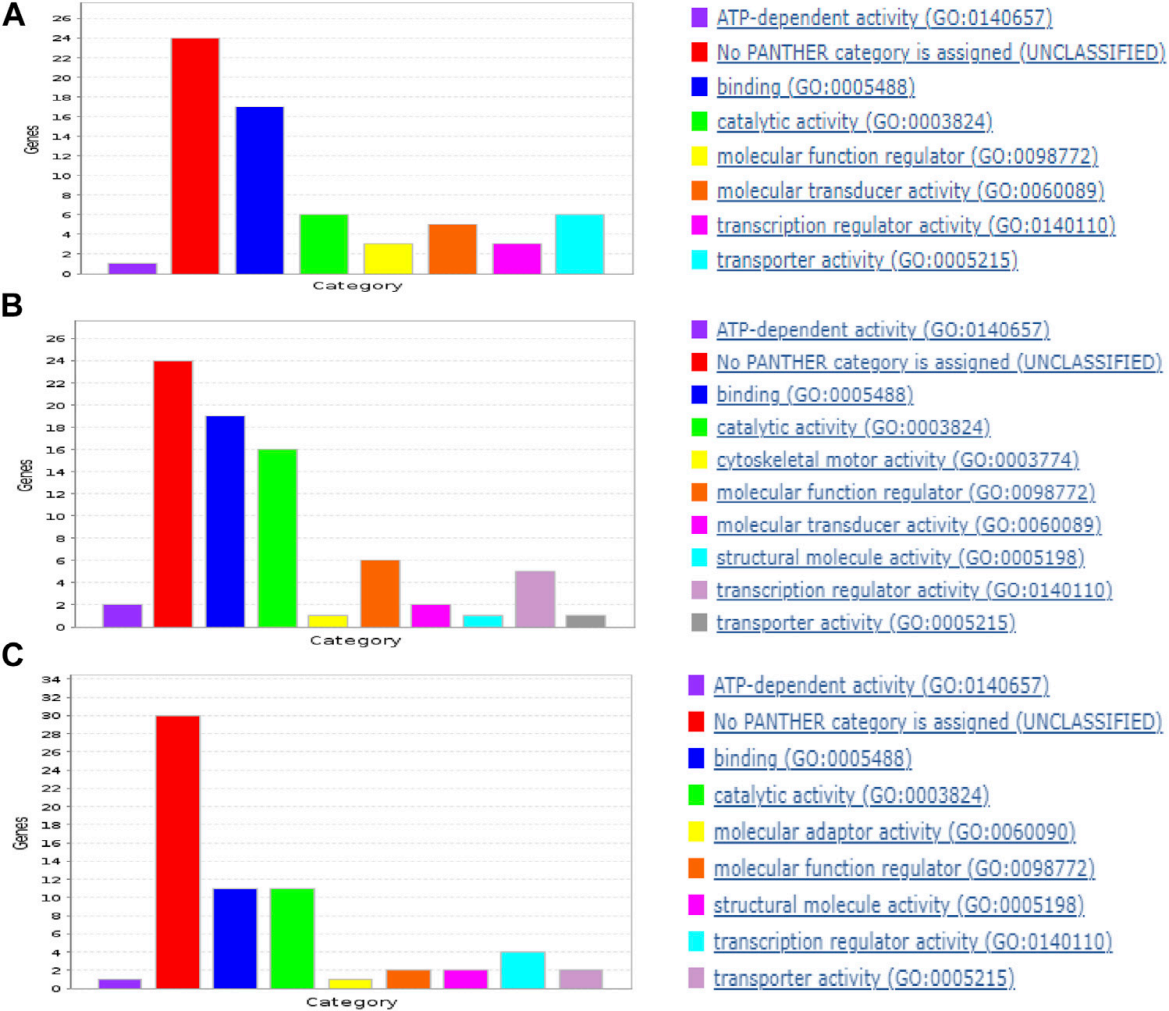
Functional classification of selected genes in **(A)** BLCA, **(B)** PAAD, and **(C)** HNSC.

In each cancer type, samples with DM were combined with the same size of samples without DM. Also, datasets from the three cancer types were combined to derive the “ALL3” group. Following pre-processing, the optimized RF algorithm, as described in the methods section was used for binary classification of the samples (i.e., DM vs. non-DM), and simultaneous gene selection (top 50) in each of the groups (Supplementary Material S2). While RF is a popular ML based gene selection technique (Díaz-Uriarte and Alvarez de Andrés, 2006; Wenric and Ruhollah, 2018; Nivedhitha et al., 2020), to the best of our knowledge, at the time of this writing, this is the first time RF is combined with this optimization method for the task of gene selection. The top 5 genes associated with metastasis in BLCA are *FKBP6, ASIC5, MAPK8IP1, F11R*, and *PABPC5*, while *CTSV, BIRC5, SERPINA7, CST2, KLHL3* are the highest ranked genes in PAAD. In HNSC, *TM4SF1, C19orf18, EXPH5, FKBP2, PTPRZ1* are the highest ranked genes, and ALL3 group had *CHRNG, CPT1B, CGREF1, GPR31, SPTBN5*. Past publications have confirmed the activities of some of these genes in the metastasis of various cancers, either as oncogene or as tumor suppressors (Table 2). There are others whose roles in cancer metastasis are yet to be explored. A functional annotation search for the selected 50 genes on pantherdb.org revealed very similar classification patterns in all of the three cancer types. Approximately 50%–60% of the genes had no known category, and the most common functional classification of those with known categories in all the cancers are protein binding, and catalytic activity (Figure 3).

**TABLE 2:** Top 10 genes selected by the ML (RF + Optimization) technique in BLCA, PAAD, HNSC, and various types of malignancies in which their involvement in metastasis have been reported in past literatures.

Gene Ranking #	Highest Ranked DM Genes in BLCA	Cancer types with Publications of Gene metastatic activities	Highest Ranked DM Genes in PAAD	Cancer types with Publications of Gene metastatic activities	Highest Ranked DM Genes in HNSC	Cancer types with Publications of Gene metastatic activities
1	**FKBP6**		**CTSV**	Breast Cancer Sereesongsaeng et al. (2020)	**TM4SF1**	Ovarian Cancer Gaoet al. (2019)
				Colorectal Cancer Wang et al. (2020)		Esophageal Cancer Xue et al. (2017)
				Lung Cancer Wang et al. (2021); Yang et al. (2022)		Pancreatic Cancer Cao et al. (2016)
						Liver Cancer Huang et al. (2016)
						Colorectal Cancer Park et al. (2016); Tang et al. (2020)
2	**ASIC5**		**BIRC5**	Colorectal Cancer Kreig et al. (2013)	**C19orf18**	
				Prostate Cancer Hennigs et al. (2020)		
				Breast Cancer Dai JB et al. (2020); Oparina et al. (2021)		
3	**MAPK8IP1**	Gastric Cancer Lu et al. (2017)	**SERPINA7**		**EXPH5**	
4	**F11R**	Breast Cancer Bednarek et al. (2020)	**CST2**	Prostate Cancer Song et al. (2021)	**FKBP2**	
		Prostate Cancer Guo et al. (2023)		Triple-Negative Breast Cancer Johnstone et al. (2018)		
		Pancreatic Cancer Zhang et al. (2022)		Gastric Cancer Zhang et al. (2020)		
		Multiple Cancers Czubak-Prowizor et al. (2022)				
5	**PABPC5**	Non-Small Cell Lung Cancer Wu et al. (2021)	**KLHL3**	Breast Cancer Mamoor. (2021)	**PTPRZ1**	Triple Negative Breast Cancer Fu et al. (2016)
		Glioma Jing et al. (2020)				Lung Cancer Chai et al. (2022)
6	**SLC5A1**	Glioblastoma Brosch et al. (2022)	**TK1**	Lung Cancer Malvin et al. (2019)	**MUC12**	Colorectal Cancer Maksuyama et al. (2010)
				Breast Cancer (Fanelli et al. (2021); He et al. (2006), Bitter et al. (2022)		Renal Cell Carcinoma Gao et al. (2020)
				Multiple Cancers Liu et al. (2017)		
7	**CCDC33**		**E2F1**	Prostate Cancer Liang et al. (2016)	**RPS10**	
				Melanoma Alla et al. (2010)		
				Breast Cancer Hollern DP et al. (2019)		
				Multiple Cancers Goody et al. (2019)		
8	**ONECUT1**	Hepatocellular Carcinoma Liu et al. (2022)	**GGH**	Gastric Cancer Terashima et al. (2017), Maezawa et al. (2020)	**RAB3D**	Osteosarcoma (Jiashi et al. (2018); Cao et al. (2019)
						Colorectal Cancer Luo et al. (2016)
						Melanoma Yang et al. (2015)
						Breast Cancer Yang et al. (2015)
						Glioma Jin et al. (2021), Tao et al. (2020)
						Non-small Cell Lung Cancer (Ma et al. (2022)
						Hepatocellular Cancer Li et al. (2020)
9	**CLC**	Gastric Cancer Gu et al. (2018); Peng a et al. (2018)	**MAGED4**	Hepatocellular Carcinoma Kanda et al. (2017)	**SH2D4A**	
		Colorectal Cancer Mu et al. (2020)		Non Small Cell Lung Cancer Ma et al. (2012)		
		Multiple cancers Xu et al. (2014)				
10	**ZNF467**	Prostate Cancer Zhang et al. (2022)	**CST6**	Breast Cancer Li et al. (2021); Jin L et al. (2012), Rivenbark et al. (2007)	**RGS16**	Chondrosarcoma Sun et al. (2015)
				Melanoma Riker AI et al. (2008)		Glioma Wang et al. (2022)
				Multiple Cancers Xu et al. (2021)		Pancreatic Cancer Kim et al. (2010); Carper MB et al. (2014)

Dataset in each group was randomly split into train and test set in a 75:25 ratio, and SVM with linear kernel, KNN, and RF models were trained on the highest ranked 30, 25, 20, 15, 10, 5, 3, 2, and 1 gene from list of selected top 50 genes. Outcomes of single instance predictions, and five-fold cross-validation on the test sets were recorded (Supplementary Material S3).

**FIGURE 4:**
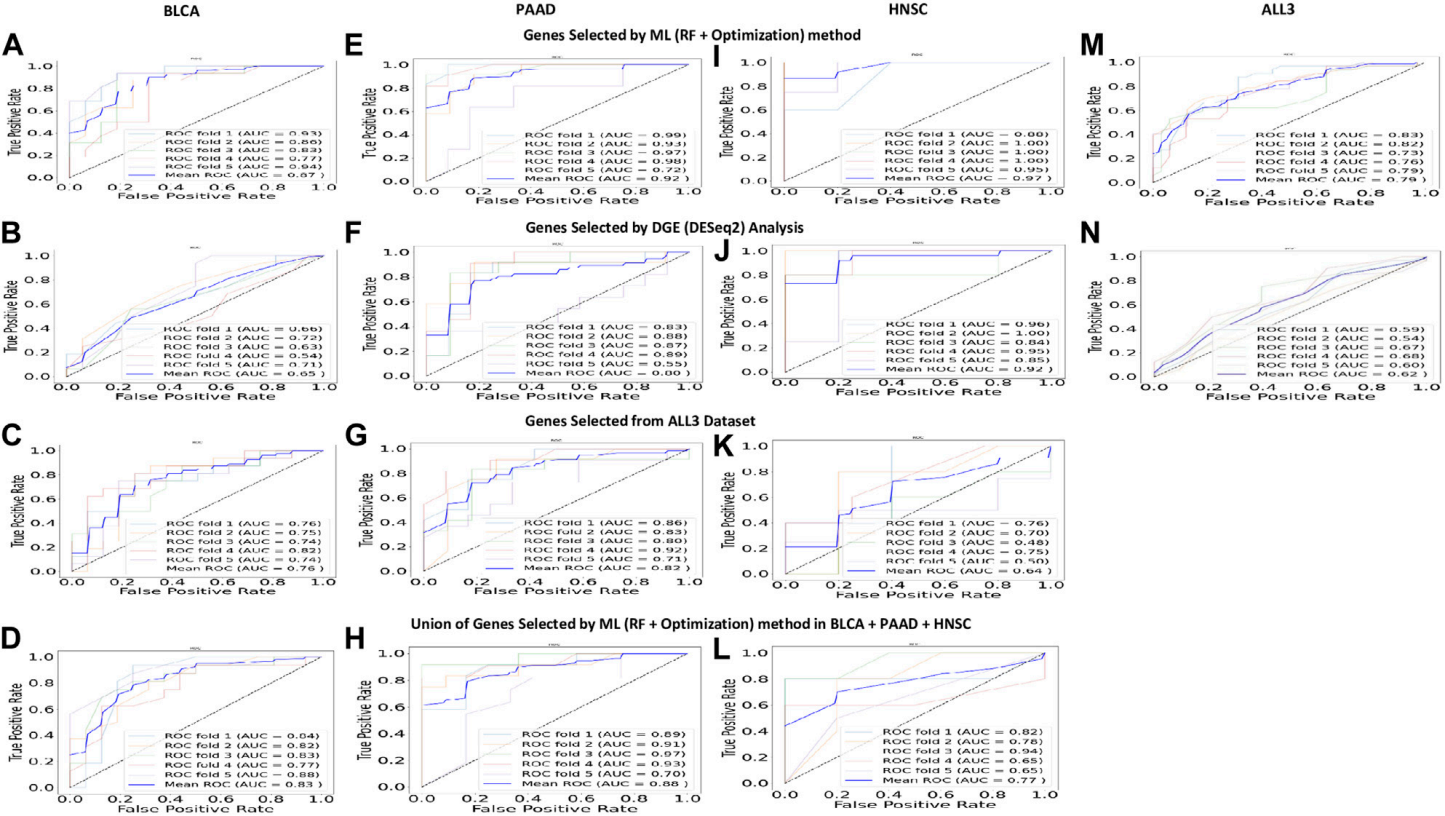
(**A, E, I, M**) Five-fold cross validation ROC curves, and mean ROC curve when 15 highest ranked genes selected by (RF + Optimization) method from each of the groups are used to predict presence or absence of DM. These are higher than other predictions within the same study group. (**B, F, J, N**) Five-fold cross validation ROC curves, and mean ROC curve when 15 highest ranked DEGs (p adjusted value = 0.05) are used to predict presence or absence of DM. **(C, G, K)** Five-fold cross validation ROC curves, and mean ROC curve when 15 highest ranked genes selected by (RF + Optimization) method from ALL3 group are used to predict presence or absence of DM in other (BLCA, PAAD, HNSC) study groups. (**D, H, L**) Five-fold cross validation ROC curves, and mean ROC curve when a union of the 15 highest ranked genes selected by (RF + Optimization) method from each of BLCA, PAAD, and HNSC groups (total = 45) are used to predict presence or absence of DM in each cancer type.

To test the strength of genes selected based on the (RF + Optimization) method as predictors of DM, a DGE analysis was carried out with the DESeq2 software package to identify DEGs between the DM and non-DM samples. With a threshold adjusted *p*-value of 0.05, the number of DEGs in BLCA, PAAD, HNSC, and ALL3 are 229, 2142, 1100, and 658 respectively. Of these 5, 39, 19, and 13 overlap with the 50 genes selected using the ML method in each of the respective groups. Presence or absence of DM was predicted in the four groups using DEGs with the lowest 30, 25, 20, 15, 10, 5, 3, 2, and 1 adjusted *p*-value as variables. In almost all cases, genes selected using the ML (RF + optimization) techniques had higher evaluation metrics (Accuracy, F1-score, AUROC) than those selected via DESeq2 DGE analysis in the task of DM prediction **(** Figure 4 **)**. Highest mean AUROC score achieved over a five-fold cross validation was 0.87, 0.92, 0.97, 0.79 in BLCA, PAAD, HNSC, and ALL3 groups respectively, compared to 0.65, 0.80, 0.92, 0.62 with DESeq2 DGE analysis, when models were trained on top 15 selected genes (Figure 4; Supplementary Material S3).

### Expression profile of genes associated with DM in primary tumors differ across cancer types

**FIGURE 5:**
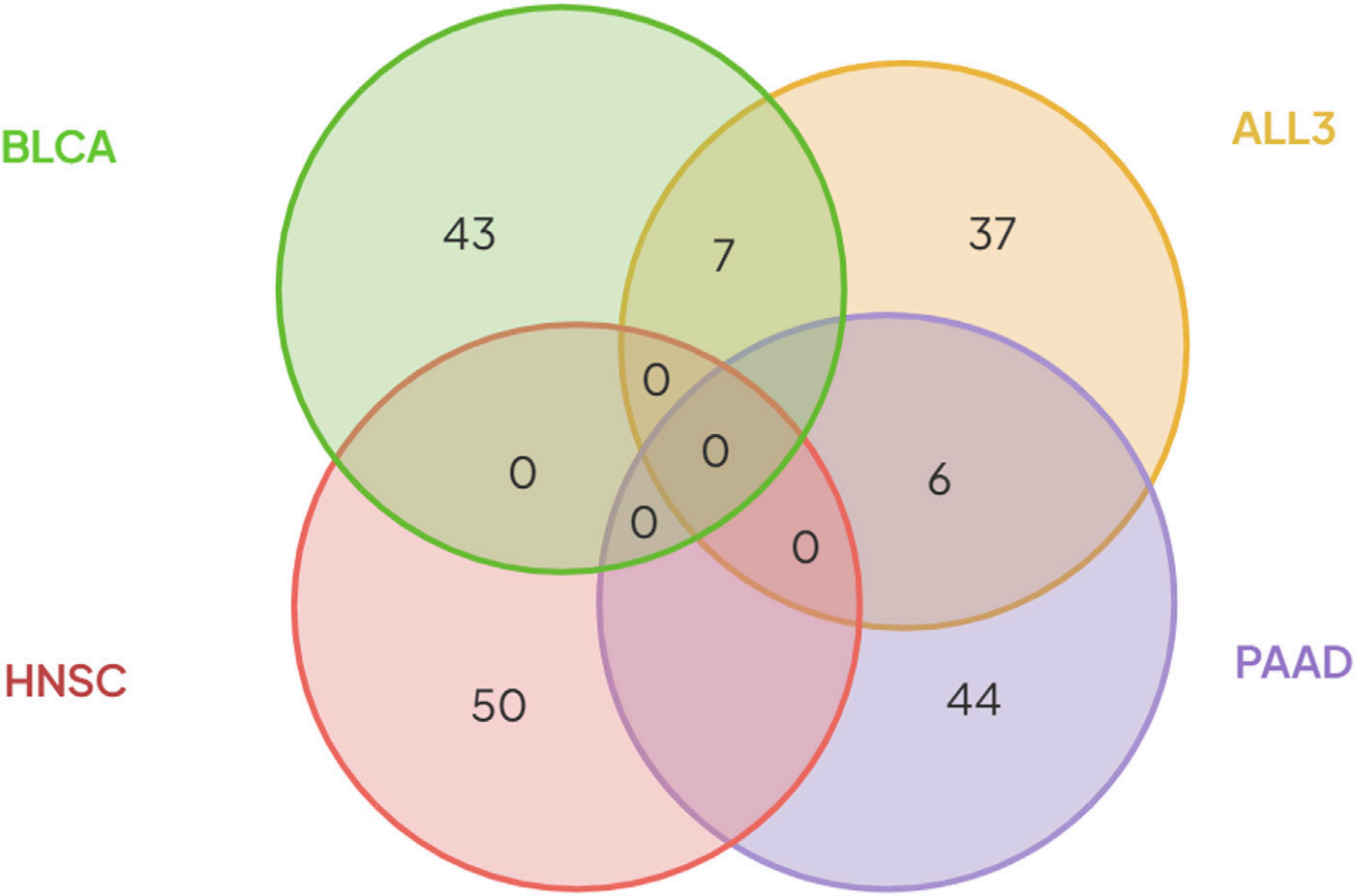
There was no overlap between genes selected using the (RF + Optimization) method in either of the three cancer types- BLCA, PAAD, and HNSC. Six genes selected in PAAD, and seven genes in BLCA were also present in the list of genes selected from the ALL3 group.

To investigate if primary tumors of different cancer types share similar gene expression profiles in metastasis, first, we looked for overlap between various combinations of the selected 50 genes in each group. There was no overlap between the different combinations of BLCA, PAAD or HNC. However, in the ALL3 group, 7 genes (*CGREF1, SPTBN5, TAS1R3, FAM241B, EL5, CSPG5*, and *MAPK8IP1*), and 6 genes (*CFAP45, CST2, BIRC5, MAGED4, CST6*, and *FAIM2*) overlap with those selected in BLCA, and PAAD respectively (Figure 5; Table 3). Also, it was observed that there was at least one member of the ***Z*** *NF* and *FKBP* gene family present within the list of selected genes in BLCA, and HNSC. Jen and Wang (2016) extensively reviewed multiple studies on the role of ZNF (Zinc Finger) gene family proteins in cancer progression, and metastasis, acting both as oncogenes, and tumor suppressors. Various studies (Fong et al., 2003; Sun et al., 2021) have also implicated members of the FKBP family in cancer metastasis.

**TABLE 3:** Number of overlapping genes between combinations of the different groups of genes selected by (RF + Optimization) method, and DGE analysis by DESeq2.

Compared Groups of Selected Genes	(RF + Optimization) method No of Overlaps Between Groups	DGE analysis (DESeq2) No of Overlaps Between Groups
BLCA | PAAD	0	32
BLCA | HNSC	0	16
PAAD | HNSC	0	160
BLCA | ALL3	7	66
PAAD | ALL3	6	305
HNSC | ALL3	0	105
BLCA | PAAD | HNSC	0	4 (**PHF21B, CALY, FGF19, and KRT81**)
BLCA | PAAD | HNSC | ALL3	0	0

Furthermore, we trained new ML (SVM, RF, and KNN) models on BLCA, PAAD, and HNSC datasets, but with genes selected from the combined dataset of the three cancer types (*i.e.*, ALL3 group) (Figure 1). These models performed lesser than in cases of predictions of DM using genes selected in individual cancer types. Highest mean AUROC score of 0.76, 0.82 and 0.64 was achieved in BLCA, PAAD, and HNSC datasets respectively against 0.87, 0.92, and 0.97 of models trained on selected genes derived from individual cancer types (Figure 4; Supplementary Material S3). To further confirm this specificity of genes in each cancer type, a union list of selected top 15 genes in the three cancer types - BLCA, PAAD, and HNSC was created. Results show a drop in mean AUROC scores from 0.87, 0.92, and 0.97 in BLCA, PAAD, and HNSC datasets respectively when 15 genes generated from each cancer type were used as variables to 0.83, 0.88, and 0.77 when the total of 45 genes in the list of unified genes were used as variables to predict DM (Figure 4; Supplementary Material S3).

### Histopathology images and clinical and genomic data for multimodal prediction of DM

DenseNet121 was trained on histopathology images from BLCA, PAAD, and ALL3 groups, after the HNSC group was dropped due to small sample size. One WSI was preprocessed per patient as described in the methods section. For each group, classification models—SVM, and MLP were trained on patient level features extracted by the trained CNN models, and prediction was carried out on the test set features (Figure 2). The image features were also combined with genomic features from the same patient to build multimodal (Image + Genomic) models.

**FIGURE 6:**
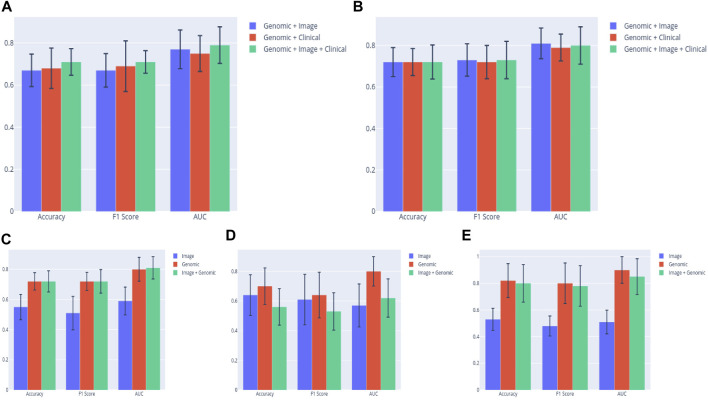
**(A)** SVM classifier clearly shows superiority of the multimodal (Clinical + Genomic + Image) model in the ALL3 dataset. **(B)** The performance of the (Clinical + Genomic + Image) model appears to be on par with the bimodal models when a MLP classifier is used. Comparing prediction metrics of the best performing bimodal (Image + Genomic) model in each group to those of unimodal models. Results from the bimodal model from the **(C)** ALL3 dataset are clearly better than those from unimodal **(D)** BLCA dataset, and **(E)** PAAD dataset.

Early results showed that metrics of multimodal (Image + Genomic) models built from the ALL3 dataset generally improved on image, or genomic unimodal models by 1–3 percent margin, with genomic data contributing more to the multimodal metrics by a large margin. Following a five-fold Monte Carlo cross-validation, a multimodal (Image + Genomic) SVM classifier produced a mean AUROC score of 0.77, while the corresponding image and genomic unimodal models produced a score of 0.58, and 0.74 respectively. The mean AUROC scores from MLP classifiers are (Image + Genomic = 0.81; Image = 0.59; Genomic = 0.80). In either BLCA or PAAD dataset, results of multimodal (Image + Genomic) models mostly improved on those of the image only models, however they were generally below that of genomic only models. The highest mean AUROC derived from a BLCA dataset multimodal (Image + Genomic) model is 0.62, and the corresponding values from image only, and genomic only model are 0.57, and 0.80 respectively. Similar pattern was seen in the PAAD dataset [Image + Genomic = 0.85; Image = 0.51; Genomic = 0.90) (Figure 6; Supplementary Material S4)]. These outcomes emphasize the importance of a large dataset for building a more robust image model when a weakly supervised training technique is employed.

Based on these early results, we carried out further experiments with the ALL3 dataset. To evaluate the effect of using a CNN model with lower layers pre-finetuned on histopathology domain images as features extractor, fully connected layers were trained on KimiaNet with the ALL3 dataset histopathology images, and features derived from the model were combined with genomic data to predict DM in the test set. There was generally no additional advantage observed in the results of KimiaNet over those from DenseNet121. Highest mean AUROC from the MLP multimodal model (KimiaNet Image + genomic data) is 0.77. Unimodal image and genomic models from the same dataset produced AUROC scores of 0.55, and 0.80 respectively. The same pattern was seen with the SVM classifier (Image + Genomic = 0.73; Image = 0.56; Genomic = 0.74) (Supplementary Material S4).

Lastly, four clinical variables—Age, T stage, N stage, and Number of lymph nodes positive by HE were concatenated with genomic, and histopathology features to train SVM, and MLP classifiers. This led to an improvement of the mean AUROC score of the Image + Genomic SVM model from 0.77 to a score of 0.79. Mean accuracy, and F1 scores also improved from 0.67 and 0.67 to 0.71 and 0.71 respectively. MLP classifier however produced a mean AUROC score of 0.80 against the score of 0.81 achieved with Genomic + Histopathology data (Figure 6). Combining clinical with genomic data improved the metrics of the clinical only models, however there was no marked improvement from metrics of the genomic only models (Figure 6; Tables 4, 5).

**TABLE 4:** Accuracy, F1, and AUROC scores derived from combinations of clinical, genomic and image data in the ALL3 dataset with a SVM classifier.

Data Type(s)	Mean accuracy	Accuracy Standard Deviation	Mean F1 Score	F1 Score Standard Deviation	Mean AUROC	AUROC Standard Deviation
Clinical	0.59	0.061	0.52	0.070	0.64	0.085
Image	0.56	0.073	0.55	0.106	0.58	0.075
Genomic	0.68	0.071	0.69	0.080	0.74	0.080
Genomic + Image	0.67	0.078	0.67	0.079	0.77	0.092
Clinical + Genomic	0.68	0.096	0.69	0.121	0.75	0.085
Genomic + Image + Clinical	**0.71**	0.063	**0.71**	0.055	**0.79**	0.087

**TABLE 5:** Accuracy, F1, and AUROC scores derived from combinations of clinical, genomic and image data in the ALL3 dataset with a MLP classifier.

Data Type(s)	Mean accuracy	Accuracy Standard Deviation	Mean F1 Score	F1 Score Standard Deviation	Mean AUROC	AUROC Standard Deviation
Clinical	0.51	0.039	0.26	0.077	0.59	0.044
Image	0.55	0.084	0.51	0.111	0.59	0.092
Genomic	0.72	0.058	0.72	0.060	0.80	0.079
Genomic + Image	**0.72**	0.070	0.73	0.078	**0.81**	0.074
Clinical + Genomic	0.72	0.065	0.72	0.080	0.79	0.065
Genomic + Image + Clinical	0.72	0.082	**0.73**	0.090	0.80	0.090

## Discussion

Given that there was barely any overlap between genes selected from the different cancer types considered and that prediction of metastasis in each of the cancer types with a union of genes selected from the three cancer types or the ALL3 group produced inferior results, we have been able to substantiate the claim that metastatic genes are more cancer type specific, rather than general, across carcinomas. As only three carcinoma types are considered in this study, studies including a larger number of carcinoma types will be needed to further solidify our findings. As shown on (Table 3), some of the selected genes have however been identified to be involved in the metastasis of multiple cancers. These further confirm the theory that even though metastatic genes tend to be specific to each carcinoma type, this specificity is based more on group of genes rather than individual genes, and that there is no single metastatic gene as have been reported in (Nguyen et al., 2022). The AUROC scores, and other metrics reported from our study, and other similar studies call for future works and development of diagnostics and therapeutics against DM to perhaps be more focused on the carcinoma type, rather than a general one size fits all approach. The *ZNF* and *FBKP* family of genes which is present in the list of selected genes in the BLCA, and HNSC datasets have been severally associated with cancer progression, and metastasis in multiple cancers (Fong et al., 2003; Jen and Wang, 2016; Sun et al., 2021), and should be investigated more for their roles in cancer metastasis.

Furthermore, with our novel combination of the RF algorithm and the described optimization technique, we have identified separate gene expression biomarkers of DM in the individual cancer types. Metrics of models built with these genes (AUC: BLCA = 0.87; PAAD = 0.92; HNSC = 0.97) outperform those built from genes selected by DESeq2 DGE analysis (AUC: BLCA = 0.65; PAAD = 0.82; HNSC = 0.92). While it is important to note that the datasets in our study are focused specifically on DM, similar studies on related tasks have been reported. Using just RF algorithm for gene selection in a breast cancer study (Yao et al., 2022), achieved an AUC of 0.52 in a metastasis and recurrence prediction task. (Wu et al., 2017), and (Qiao et al., 2020) achieved AUC scores of 0.71 and 0.84 respectively when genes derived from DESeq2 DGE analysis, and Boruta algorithm in the latter were combined with clinical data for prediction of lymph node metastasis in HNSC. In predicting metastasis status in pancreatic cancer samples (Xue et al., 2021), achieved a highest AUC score of 0.72 using DEG identified by the edgeR package. A 51 gene signature produced an AUC score of 0.82 in prediction of lymph node metastasis in bladder cancer as reported by (Seiler et al., 2016).

The reported AUC scores from our study were derived from the highest ranked 15 genes out of the initially selected 50. Perhaps a protein-protein interaction analysis including the initial 50 genes could lead to selection of fewer genes with higher relevance than the ranking we have employed here, which may in turn lead to improved prediction metrics. The biomarkers discovered in each cancer type using the strict ML approach we have employed should be further investigated for their potential as diagnostic indicators, and as basis for development of new therapies against cancer metastasis.

Our results indicate that multimodal data (Genomic + Clinical + Histopathology) provides a predictive advantage over either of the unimodal data types in the task of DM detection, however the contribution of only gene expression data is highest by a wide margin. Similar studies have acknowledged the superiority of multimodal data for prediction of medical diagnosis and various outcomes in cancer patients (Mobadersany et al., 2018; Keihl et al., 2021; Chen et al., 2022), however some studies have shown that this is not always the case (Brinker et al., 2021; Vale-Silva and Rohr, 2021). Past multimodal studies on prediction of metastasis from histopathology images have mostly combined clinical data with histopathology images. This combination used in prediction of nodal metastasis led to an improved AUC score of 74% in a study by (Keihl et al., 2021). In contrast, there was a 0.2% decrease from the only image AUC score of 61.8% reported by (Brinker et al., 2021) when clinical, and cellular data were included. Results from our study also suggest the importance of a larger dataset of images when a weakly supervised technique is employed as noted with the superior metrics of the multimodal models including histopathology images in the ALL3 dataset compared to BLCA, and PAAD dataset. A recent study by (Yao et al., 2022) reported a higher accuracy in model built from only genomic data compared to that built on combined genomic, and histopathology data for prediction of metastasis and recurrence in breast cancer. However, an AUC score of 0.75 was achieved when gene expression, histopathology images and clinical data were combined.

The highest mean AUC score of 0.79 derived from the SVM classifier in our study was from multimodal combination of gene expression, clinical, histopathology data, while the score of 0.81 derived from the MLP classifier was obtained from a genomic + histopathology image model. Overall, our results indicate that combining clinical, genomic, and histopathology image data increases the prediction metrics for DM, however genomic data alone is a strong contributor.

## Data Availability

The original contributions presented in the study are included in the article/Supplementary Material, further inquiries can be directed to the corresponding author.
