# Supplementary material for: Multimodal AI for prediction of distant metastasis in carcinoma patients
Source: Front Bioinform. 2023 May 9;3:1131021. doi: 10.3389/fbinf.2023.1131021 (PMC10203594; doi:10.3389/fbinf.2023.1131021)
Supplement: Supplementary file 3 [file Table5.DOCX]

| **Cancer Types** | **No of Cases with Recorded Distant Metastasis** | **No of Cases with Diagnostic Slides** | **No of Cases with Gene Expression Data** |
| --- | --- | --- | --- |
| HNC (Head and Neck Cancer) | 24 | 24 (23) | 24 (23) |
| BRCA (Breast Invasive Carcinoma) | 28 | 28 (25) | 28 (28) |
| BLCA (Bladder Urothelial Carcinoma) | 82 | 82 (74) | 82 (80) |
| LIHC (Liver Hepatocellular Carcinoma) | 31 | 31 (30) | 31 (30) |
| COAD (Colon Adenocarcinoma) | 9 | 9 (8) | 9 (8) |
| Lungs (Lungs Adenocarcinoma) | 10 | 10 (8) | 10 (9) |
| LUSC (Lungs Squamous Cell Carcinoma) | 14 | 14(12) | 14 (14) |
| STAD (Stomach Adenocarcinoma) | 26 | 26 (22) | 26 (23) |
| SKCM (Skin Cutaneous Melanoma) | 92 | 92 (89) | 92 (92) |
| PAAD (Pancreatic Adenocarcinoma) | 59 | 59 (58) | 59 (58) |
| THCA (Thyroid Carcinoma) | 12 | 12 (12) | 12 (12) |
